# The Possible Transmission and Potential Enterotoxicity of *Bacillus cereus* on Lettuce Farms in Five Chinese Provinces

**DOI:** 10.3389/fmicb.2021.746632

**Published:** 2021-10-01

**Authors:** Yang Qu, Chao Wei, Xiaohang Dai, Yalong Bai, Xin Zhao, Qingkuo Lan, Wenbo Wang, Yuanjuan Wu, Min Gao, Weihao Tang, Changyan Zhou, Yujuan Suo

**Affiliations:** ^1^Laboratory of Quality and Safety Risk Assessment for Agro-Products of Ministry of Agriculture and Rural Affairs, Institute for Agro-Food Standards and Testing Technology, Shanghai Academy of Agricultural Sciences, Shanghai, China; ^2^Shanghai Co-Elite Agro-Food Testing Service Co., Ltd., Shanghai, China; ^3^Laboratory of Quality and Safety Risk Assessment for Agro-Products of Ministry of Agriculture and Rural Affairs, Institute of Quality Standard and Testing Technology for Agro-Products of Sichuan Academy of Agricultural Sciences, Chengdu, China; ^4^Institute of Germplasm Resources and Biotechnology, Tianjin Academy of Agricultural Sciences, Tianjin, China; ^5^Laboratory of Quality and Safety Risk Assessment for Agro-Products of Ministry of Agriculture and Rural Affairs, Institute of Agricultural Standards and Testing Technology for Agri-Products, Shandong Academy of Agricultural Sciences, Jinan, China; ^6^Laboratory of Quality and Safety Risk Assessment for Agro-Products Processing of Ministry of Agriculture and Rural Affairs, Institute of Food Science and Technology, Chinese Academy of Agricultural Sciences (CAAS), Beijing, China

**Keywords:** *Bacillus cereus*, lettuce farm, multilocus sequence typing (MLST), tracing analysis, enterotoxicity

## Abstract

*Bacillus cereus* is a well-characterized human pathogen that produces toxins associated with diarrheal and emetic foodborne diseases. To investigate the possible transmission of *B. cereus* on lettuce farms in China and determine its enterotoxicity, (I) a total of 524 samples (lettuce: 332, soil: 69, water: 57, manure: 57, pesticide: 9) were collected from 46 lettuce farms in five Chinese provinces, (II) multilocus sequence typing (MLST) was used to classify *B. cereus* isolates and for trace analysis, and (III) the presence of toxin genes and enterotoxins (Hbl and Nhe) was detected in 68 strains. The results showed that one hundred and sixty-one lettuce samples (48.5%) tested positive for *B. cereus* at levels ranging from 10 to 5.3 × 10^4^ CFU/g. Among the environmental sample categories surveyed, the highest positive rate was that of the pesticide samples at 55.6%, followed by soil samples at 52.2% and manure samples at 12.3%. Moreover, one hundred isolates of *B. cereus* yielded 68 different sequence types (STs) and were classified into five phylogenetic clades. Furthermore, Nhe toxin genes (*nheA*, *nheB*, *nheC*) were broadly distributed and identified in all 68 strains (100%), while Hbl toxin genes (*hblA*, *hblC*, *hblD*) were present in 61 strains (89.7%), *entFM* was detected in 62 strains (91.2%), and *cytK* was found in 29 strains (42.6%). All strains were negative for *ces*. As for the enterotoxin, Nhe was observed in all 68 isolates carrying *nheB*, while Hbl was present in 76.5% (52/68) of the strains harboring *hblC.* This study is the first report of possible *B. cereus* transmission and of its potential enterotoxicity on lettuce farms in China. The results showed that soil and pesticides are the main sources of *B. cereus* on lettuce farms in China, and the possible transmission routes are as follows: soil-lettuce, manure-lettuce, pesticide-lettuce, manure-soil-lettuce, and water-manure-soil-lettuce. Furthermore, the *B. cereus* isolates, whether from lettuce or the environment, pose a potential risk to health.

## Introduction

The World Health Organization recommends a daily intake of 400 g of fresh vegetables for improved health (Park et al., 2018). Lettuce is the most widely consumed fresh vegetable and is usually eaten raw with no or minimal processing, increasing the occurrence of lettuce-related foodborne outbreaks, which have gained attention among government agencies, industries, and the public (Bozkurt et al., 2021). Raw lettuce has been reported to harbor foodborne pathogens, such as *Bacillus cereus*, *Escherichia coli* O157:H7, *Salmonella*, and *Listeria monocytogenes* (Abadias et al., 2008; Park et al., 2018; Yu et al., 2019). A total of 597 outbreaks caused by *B. cereu*s toxins involving 6,221 cases were reported in European Member States (MSs) from 2014 to 2016 due to the diarrhea and emesis (vomiting) caused by *B. cereus* (Osimani et al., 2018). Thus, the safety of *B. cereus* strains in lettuce should be considered.

*Bacillus cereus* is a spore-forming gram-positive species (Ceuppens et al., 2013) that is distributed among seven phylogenetic clades with nine species (Guinebretière et al., 2008), including (I) *B. cereus sensu stricto* and *B. cytotoxicus*, which cause foodborne illness (Dierick et al., 2005; Guinebretière et al., 2008); (II) *B. weihenstephanensis* and *B. mycoides*, which cause food spoilage (Meer et al., 1991); (III) *B. anthracis*, which causes anthrax in both humans and animals (Ivanova et al., 2003); (IV) *B. thuringiensis*, which is used as an insecticide in agriculture (Höfte and Whiteley, 1989); (V) *B. pseudomycoides*, which is considered a non-pathogenic environmental microorganism; (VI) *B. toyonensis*, which has recently been recognized as a putative probiotic species (Jiménez et al., 2013); and (VII) *B. wiedmannii*, which is a psychrotolerant cytotoxic species (Miller et al., 2016). Therefore, it is difficult to identify *B. cereus* isolates, and multilocus sequence typing (MLST) is recommended in combination with traditional methods to differentiate diverse species of *B. cereus* (Jung et al., 2011; Maiden et al., 2013; Otlewska et al., 2013; Castiaux et al., 2014; Zhuang et al., 2019).

Farm-to-fork supply chains are considered to be the source of *B. cereus* in lettuce, including field production, harvest, processing, packaging, transportation, retail, and home storage (Pang et al., 2017), and farm environments are the core source (Drewnowska et al., 2020). Moreover, MLST is a powerful method for tracing analysis based on genetic evolution, which can be determined by comparative analysis of alleles (Forsythe et al., 2014; Hammerum et al., 2015). Processing environments and packing areas have been found to be the sources most likely to be associated with *B. cereus* contamination in powdered infant formula production (Zhuang et al., 2019). The transmission of *B. cereus* in lettuce farms must be detected and monitored to promote food safety and human health.

In this study, the prevalence of *B. cereus* in lettuce and farm environments distributed in China was described. Genetic methods were applied to examine the phylogenetically diverse *B. cereus* isolates from lettuce and farm environments, and their associations were examined to identify the possible transmission of *B. cereus* in lettuce farms. In addition, the safety of *B. cereus* isolates was investigated by identifying virulence factors and enterotoxins from lettuce and in farm environments.

## Materials and Methods

### Sample Collection and Isolation

**FIGURE 1:**
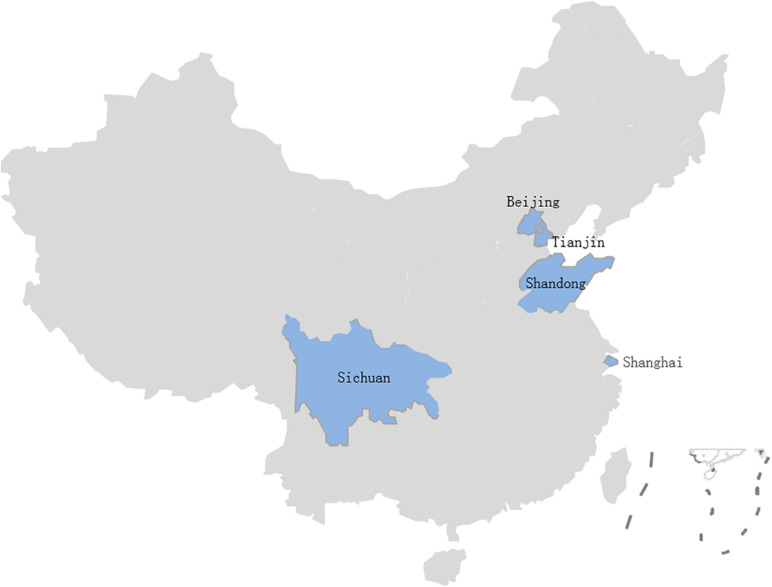
*B. cereus* isolates from 46 lettuce farms in five Chinese provinces.

A total of 524 samples were collected from 46 lettuce farms in five Chinese provinces from April 2019 to November 2020 (Figure 1). These samples were classified as lettuce (*Lactuca sativa L.*) (*n* = 332), soil (*n* = 69), water (*n* = 57), manure (*n* = 57), and pesticide (*n* = 9) samples; more details about the samples can be found in Table 1. The samples were transported to the laboratory in a cold box (4°C) within 2 h.

**TABLE 1:** Prevalence and contamination level of *B. cereus* in different samples among 46 lettuce farms.

**Sample types**	**Contamination rate^a^**	**Contamination level (CFU/g)**			
		** < 10**	**10 ≤ value < 10^2^**	**10^2^ ≤ value < 10^4^**	**10^4^ ≤ value < 10^5^**
Lettuce	48.5% (161/332)	51.5% (171/332)	22.0% (73/332)	25.9% (86/332)	0.6%(2/332)
Soil	52.2% (36/69)	47.8% (33/69)	10.1% (7/69)	34.8% (24/69)	7.3% (5/69)
Water	1.8% (1/57)	98.3% (56/57)	1.8% (1/57)	0.0% (0/57)	0.0% (0/57)
Manure	12.3% (7/57)	87.7% (50/57)	5.3% (3/57)	7.0% (4/57)	0.0% (0/57)
Pesticide	55.6% (5/9)	44.5% (4/9)	33.3% (3/9)	22.2% (2/9)	0.0% (0/57)
Total	40.0% (210/524)	60.0% (314/524)	16.6% (87/524)	22.1% (116/524)	1.3% (7/524)

*^*a*^Contamination rate = number of positive samples/total samples.*

The *B. cereu*s strains were isolated in accordance with GB4789.14-2014, the National Food Safety Standard for Food Microbiological Examination used in China. Genomic DNA was extracted from overnight cultures of *B. cereu*s isolates in Luria-Bertani (LB) broth using a TIANamp Bacteria DNA Extraction Kit (Tiangen Biotech, Beijing, China) according to the manufacturer’s protocol for gram-positive bacteria.

### Phylogenetic Study

Isolates were characterized by using seven housekeeping genes (*glp*, *gmk*, *ilvD*, *pta*, *pur, pycA*, and *tpi*) with different primers and conditions (Table 2), which are provided in the *B. cereus* PubMLST database^1^. The PCR products were sequenced by Sangon Biotech (Shanghai, China) and submitted to the *B. cereus* PubMLST database to obtain the allele number. By combination of allele numbers for all seven housekeeping genes, a sequence type (ST) clonal complex could be obtained. New allele sequences and STs were submitted to the *B. cereus* PubMLST database and assigned by the MLST website administrator.

**TABLE 2:** Primers used in the study.

**Primer**	**Sequence (50–30)**	**Target fragment length (bp)**	**Annealing temperature (°C)**	**References**
*glpF*-F	GCG TTT GTG CTG GTG TAA GT	549	59	https://pubmlst.org/bcereus/
*glpF*-R	CTG CAA TCG GAA GGA AGA AG			
*gmk*-F	ATT TAA GTG AGG AAG GGT AGG	600	56	
*gmk*-R	GCA ATG TTC ACC AAC CAC AA			
*ilvD*-F	CGG GGC AAA CAT TAA GAG AA	556	58	
*ilvD*-R	GGT TCT GGT CGT TTC CAT TC			
*pta*-F	GCA GAG CGT TTA GCA AAA GAA	576	56	
*pta*-R	TGC AAT GCG AGT TGC TTC TA			
*pur*-F	CTG CTG CGA AAA ATC ACA AA	536	56	
*pur*-R	CTC ACG ATT CGC TGC AAT AA			
*pycA*-F	GCG TTA GGT GGA AAC GAA AG	550	57	
*pycA*-R	CGC GTC CAA GTT TAT GGA AT			
*tpi*-F	GCC CAG TAG CAC TTA GCG AC	553	58	
*tpi*-R	CCG AAA CCG TCA AGA ATG AT			
*hblA*-F	AAGCAATGGAATACAATGGG	1,154	56	Guinebretière et al., 2002
*hblA*-R	ACGAATGTAATTTGAGTCGC			
*hblC*-F	GATACTCAATGTGGCAACTGC	740	58	
*hblC*-R	TTGAGACTGCTCGTCTAGTTG			
*hblD*-F	AATCAAGAGCTGTCACGAAT	411	58	
*hblD*-R	CACCAATTGACCATGCTAAT			
*nheA*-F	GTTAGGATCACAATCACCGC	755	56	
*nheA*-R	ACGAATGTAATTTGAGTCGC			
*nheB* -F	CTATCAGCACTTATGGCAG	754	54	
*nheB* -R	ACTCCTAGCGGTGTTCC			
*nheC* -F	CGGTAGTGATTGCTGGG	564	58	
*nheC*-R	CAGCATTCGTACTTGCCAA			
*entFM*-F	AAAGAAATTAATGGACAAACTCAAACTCA	596	58	Guinebretière et al., 2002
*entFM*-R	GTATGTAGCTGGGCCTGTACGT			
*cytK*-F	GTAACTTTCATTGATGATCC	505	48	Stenfors and Granum, 2001
*cytK*-R	GAATACTAAATAATTGGTTTCC			
*ces*-F	GGTGACACATTATCATATAAGGTG	1,271	58	Ehling-Schulz et al., 2005
*ces*-R	GTAAGCGAACCTGTCTGTAACAACA			

The phylogenetic study included all the isolates and nine references obtained from the NCBI database^2^ (*B. anthracis* Ames Ancestor, *B. cereus* ATCC 1457, *B. cytotoxicus* NVH 391-98, *B. mycoides* DSM 2048, *B. pseudomycoides* DSM 12442, *B. thuringiensis* ATCC 10792, *B. toyonensis* BCT-7112, *B. weihenstephanensis* WSBC 10204, *B. wiedmannii* FSL W8-1069) (Miller et al., 2018). The phylogenetic tree was constructed using the neighbor-joining (NJ) method in Molecular Evolutionary Genetic Analysis (MEGA-X) based on the concatenated sequences (2,829 bp) of the seven housekeeping genes (Kumar et al., 2018). In addition, 1,000 bootstrap replicates were used for branch quality.

### Tracing Analysis

To analyze the relationship between different STs and sample sources, a minimum spanning tree was constructed with PHYLOViZ 2.0 software (Instituto de Microbiologia, Portugal) (Francisco et al., 2012) with the goeBURST algorithm and 1,000 bootstrap resamplings (Feil et al., 2004).

### Detection of Virulence Genes

Nine virulence genes, namely, *hblA*, *hblC*, *hblD*, *nheA*, *nheB*, *nheC*, *entFM*, *cytK*, and *ces*, were identified according to a previous study (Stenfors and Granum, 2001; Guinebretière et al., 2002; Ehling-Schulz et al., 2005; Kim et al., 2011), and the primers and conditions are listed in Table 2. The 20 μL PCR mixture consisted of 10 μL of Taq^TM^ PCR Premix (Takara, China), 1 μL of diluted DNA, and 0.2 mM each primer.

### Detection of the Enterotoxins

A Duopath^®^ Cereus Enterotoxins kit (Merck, Kenilworth, NJ, United States) was used to detect Hbl (the lytic component L2, a subunit of toxin Hbl) and NheB (the binding component of the Nhe toxin), the detection limits for which were 20.0 and 6.0 ng/mL, respectively. Isolates were maintained on brain heart infusion (BHI; Co. CM1135, Oxoid, Hampshire, United Kingdom) agar plates at 4°C. Single colonies of bacteria were aseptically picked and cultured in 1 mL of CGY broth (with 1% glucose) and incubated for 4 h at 37°C. For testing, the cultures and Duopath^®^ kit were both cooled to room temperature (20°C), and 150 μL of culture was pipetted into the circular sample port on the Duopath^®^ kit. The results could be observed 30 min after applying the culture to the kit at room temperature (Krause et al., 2010).

## Results

### Prevalence Analysis of *B. cereus* in Lettuce and Farm Environments

In this study, *B. cereus* was detected in 210 of the 524 (40.0%) samples from 46 lettuce farms (Table 1) distributed in five Chinese provinces (Figure 1). The positivity rates of *B. cereus* were 48.5% (161/332) for lettuce, ranging from 10 to 5.3 × 10^4^ lg CFU/g, and the farm environment also contained *B. cereus*. Of these environmentally positive samples, the highest frequency of *B. cereus* was found in pesticides (55.6%), followed by soil (52.2%), manure (12.3%), and water (1.8%). Enumeration of *B. cereus* showed that 60.0% of the lettuce farm samples had counts below 10 CFU/g, 16.6% had counts in the range from 10–10^2^ CFU/g, 22.1% had counts in the range from 10^2^–10^4^ CFU/g, and 1.3% had counts above 10^4^ CFU/g. High counts (>10^4^ CFU/g) were most frequently found in soil and lettuce.

### Genetic Structure of *B. cereus* Populations

**FIGURE 2:**
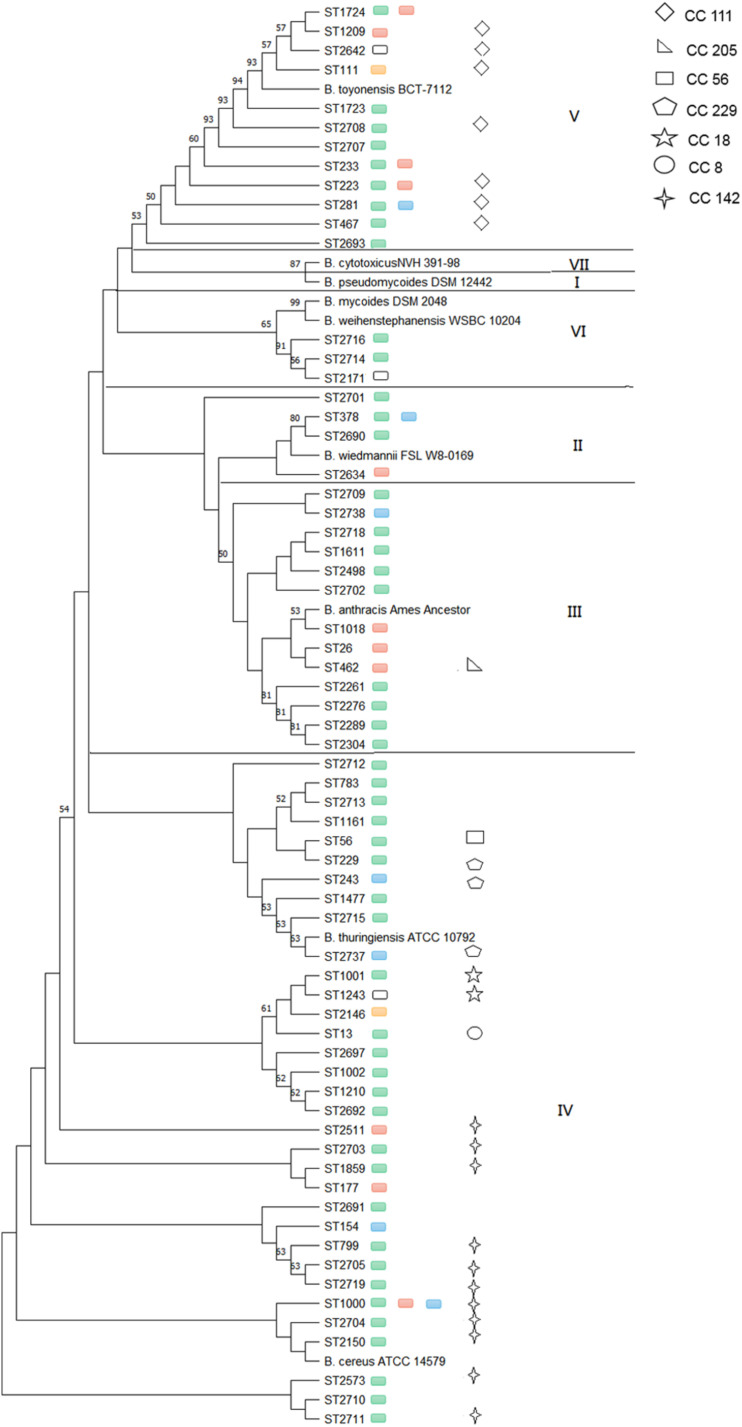
Phylogenetic relatedness of *B. cereus* strains from different provinces within particular phylogenetic groups. Green boxes represent isolates from Shanghai, red boxes represent isolates from Beijing, blue boxes represent isolates from Tianjin, yellow boxes represent isolates from Shandong, and white boxes represent isolates from Sichuan.

Considering the effect of source, location and sampling time, strains collected from the same lettuce or soil on the same farm during different sampling seasons were removed, and only 100 isolates that from lettuce, soil, pesticides, manure and water were selected for genetic structure analysis. A total of 65 different STs and seven clonal complexes (CCs) were identified among the isolates (Figure 2). Thirty-seven isolates were assigned to 31 new STs. Forty-seven of the 65 (72.3%) STs included only one isolate; however, the remaining 18 STs included more than one isolate. The most frequent ST was ST1000, comprising five isolates from lettuce and soil samples, which were found in Shanghai, Beijing, and Tianjin provinces.

The phylogenetic study was based on MLST, which clustered the STs into five phylogenetic clades (II to VI) (Guinebretière et al., 2008), and nine additional *B. cereus* clade species were included in phylogenetic analyses to serve as a reference (Miller et al., 2018). The most common clade was clade IV (50.8%), followed by clade III (20.0%), clade V (18.5%), clade II (6.2%), and clade VI (4.6%). Clade IV included both *B. cereus sensu stricto* ATCC 14579^*T*^ (ST4) and *B. thuringiensis* ATCC 10792^*T*^ as well as 33 STs obtained from five provinces that consisted of five CCs: CC8, CC18, CC56, CC229, and CC142. Frequent types of ST1000 and CC142 (11 STs) were observed in this clade. Clade III with 13 STs, in addition to the *B. anthracis* Ames ancestor, was represented by Shanghai (69.2%), Beijing (23.1%), and Tianjin (7.7%). Only CC205 (1 ST) was included in this clade. Clade V, with the *B. toyonensis* BCT-7112^*T*^ reference strain (ST111), contained 12 STs in five provinces and strains mainly from Shanghai (56.2%) and Beijing (25.0%), while other geographic locations were represented by less than 7.0% of isolates. ST1724, ST233, ST223, and ST281 were found in two different provinces, and isolates in clade V were clustered together in CC111 (7 STs). Clade II included the *B. wiedmannii* FSL W8-0169^*T*^ (ST1081) reference strain and four STs from Shanghai, Beijing, and Tianjin, while ST378 was found in both Shanghai and Tianjin. Clade VI contained both the *B. mycoides* DSM 2048^*T*^ and *B. weihenstephanensis* WSBC 10204^*T*^ reference strains as well as three STs in the Shanghai and Sichuan provinces. The isolates in clades II and VI were singletons. None of the *B. cereus* isolate strains were clustered with *B. pseudomycoides* DSM 12442^*T*^ within clade I or with *B. cytotoxicus* NVH 391-98^*T*^ within clade VII.

### Tracing Analysis

**FIGURE 3:**
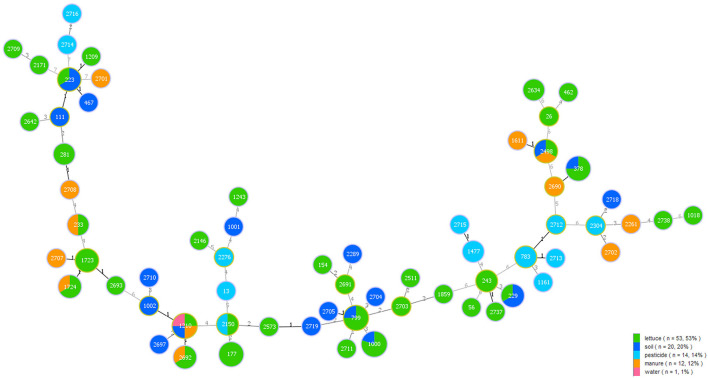
Minimum spanning tree analysis of 100 *B. cereus* isolates collected from different sample categories on lettuce farms. The size of each circle indicates the number of STs. Different sample categories are represented by different colors, and the size of each color block represents the number of STs in that source.

Among the 100 *B. cereus* isolates, 53 were collected from lettuce, 19 from soil, 14 from pesticide, 13 from manure, and 1 from water. A minimum spanning tree-like structure was drawn to show the link between the sample sources and different STs of *B. cereus* (Figure 3). The ST type in lettuce was associated with the environmental source, and there was no crossover between environmental samples. Isolates from lettuce and soil had seven identical STs, while manure had five STs, identical to lettuce. Pesticide and water also had one ST, consistent with lettuce. Five STs were associated with lettuce and soil, namely, ST223, ST229, ST378, ST799, and ST1000, while three STs (ST233, ST1724, and ST2692) were commonly recovered from lettuce and manure. Only ST2150 originated from lettuce and pesticide. Moreover, there were other STs from additional source categories: one major ST2498 was a prevalent ST type in lettuce, soil and manure. In addition, ST1210 was found in lettuce, soil, manure, and water.

### Enterotoxic Potential of *B. cereus*

**FIGURE 4:**
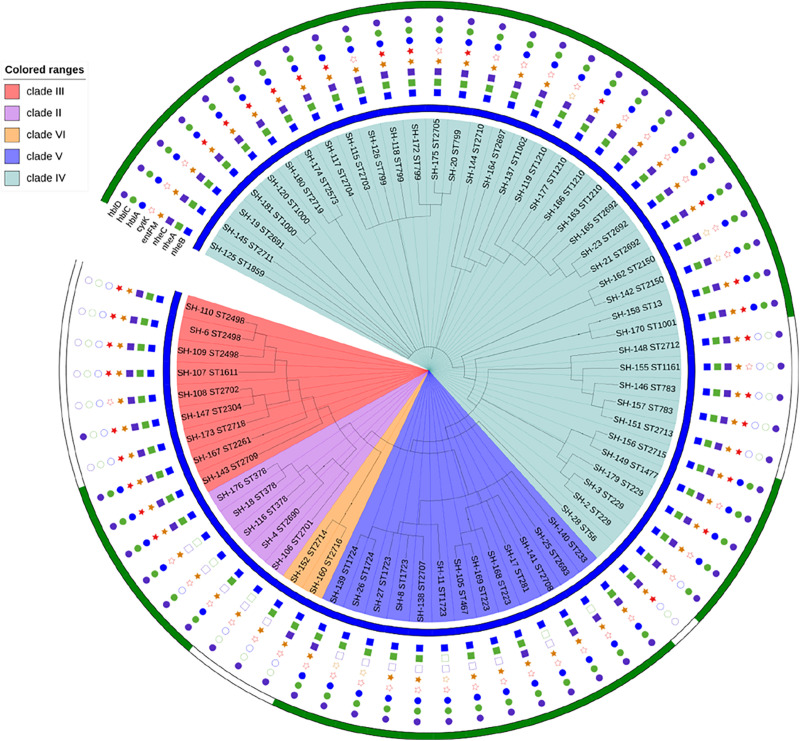
Enterotoxic potential of 68 *B. cereus* strains. The blue color strip represents Nhe (enterotoxin), while green represents Hbl (enterotoxin). The blue square represents *NheB* (gene), the green square represents *nheA* (gene), the purple square represents *nheC* (gene), the orange star represents *entFM* (gene), the red star represents *cytK* (gene), the blue circle represents *hblA* (gene), the green circle represents *hblC* (gene), and the purple circle represents *hblD* (gene).

Sixty-eight strains were selected from lettuce farms, corresponding to sources and phylogenetic groups, for further enterotoxic potential (Figure 4). All isolates were tested *via* PCR for the presence of key toxin genes. (1) The most frequently distributed genes were those encoding the enterotoxin Nhe, namely, the *nheB*, *nheA* and *nheC* genes, which were detected in 100.0, 91.2, and 80.9% of the tested strains, respectively. While these *nheABC* genes (the strains were positive for *nheA*, *nheB*, and *nheC* at the same time) genes were all detected in 54 isolates in clades III and IV, *nheA* and *nheC* were detected less frequently in clades II, VI, and VI (23.1%∼80.0%). Isolates from lettuce, soil, water, manure, and pesticides all harbored the Nhe toxin genes (Figures 3, 4). (2) Genes encoding Hbl (*hblA*, *hblC*, and *hblD*) were detected in 89.7% of the tested strains, and sixty-one Hbl PCR-positive isolates were distributed in clade II (100.0%), clade V (100.0%), clade IV (100.0%), clade IV (97.4%), and clade III (33.3%). *hblD* was the most commonly detected Hbl toxin genes (89.7%), while *hblA* and *hblC* were detected individually in 76.5 and 73.5% of the isolates, respectively. Fifty strains harbored *hblACD* (the strains were positive for *hblA*, *hblC*, and *hblD* at the same time). Among the sources, 100.0% of the water and pesticides isolates (1/1 water isolate), 92.0% of the lettuce isolates, 88.9% of the soil isolates, and 75.0% of the manure isolates harbored the Hbl toxin genes. (3) The *entFM* gene, encoding enterotoxin FM, was also broadly distributed (91.2%). All six PCR-negative isolates were present in clade IV and clade V. In addition, the *entFM* gene was detected in 100% of the isolates from all sources except lettuce and pesticides. Thus, the positive detection rate was still high in lettuce and pesticides, with values of 80.0 and 91.7%, respectively. (4) The *cytK* gene, encoding cytotoxin K, was detected in 42.6% of the strains. All PCR isolates positive for *cytK* represented clades III (88.9%), IV (51.3%), and II (20.0%). The strains harboring the *cytK* gene were isolated from all five sources. The highest percentage of *cytK* detected was 100.0%, which was observed in water (1/1 isolate), followed by 58.3% in pesticides, 50.0% in soil, 36.0% in lettuce, and 25.0% in manure. (5) The *ces* gene, encoding emetic toxin, known as cerulide synthetase, was not detected in the isolates.

All *B. cereus* isolates used in this study carried at least 3 of the 8 enterotoxin genes tested. The distribution of virulence genes was divided into 17 different profiles. Eighteen isolates possessed all eight virulence genes, which was one of the main gene profiles. The other main gene profile (18/68 of all isolates) was *hblA*-*hblC*-*hblD*-*nheA*-*nheB*-*nheC*-*entFM*. Only one isolate, SH-106 (ST2701), harbored the smallest virulence gene profile (*nheB*-*hblD*-*entFM*).

Nhe and Hbl, the most notable enterotoxins, were analyzed to obtain a broader view of the potential *B. cereus* enterotoxicity (Figure 4). Nhe was observed in the 68 isolates isolated from five sources and all phylogenetic groups, while Hbl was present in 76.5% (52/68) of the strains that were clustered into groups, with the exception of clade VI. The percentages of strains harboring Hbl were 100.0% in clade V, 84.6% in clade IV, 80.0% in clade II, and 22.2% in clade III. Among the sources, 100.0% of the water isolates (1/1), 92.0% of the lettuce isolates, 88.9% of the soil isolates, 66.7% of the manure isolates, and 33.3% of the pesticide isolates harbored Hbl. Figure 4 also reveals the relationship between virulence genes and enterotoxins: (1) Nhe was expressed when the *nheB* gene was present, and (2) Hbl was detected when the strain harbored the *hblC* gene.

## Discussion

*Bacillus cereus* is widely distributed around the world, and some studies have evaluated *B. cereus* in vegetables (Gdoura-Ben Amor et al., 2018; Park et al., 2018; Yu et al., 2019), while few studies have examined it in planting environments (Drewnowska et al., 2020). In this study, we determined the occurrence of *B. cereus* on lettuce farms in five Chinese provinces and determined the possible contamination pathways. Moreover, the potential of the isolates to cause foodborne disease was evaluated based on their production of diarrheal and emetic toxins.

The overall prevalence (48.5%) of *B. cereus* observed in the current study was somewhat less than previous research (57.7%) about markets in China (Yu et al., 2019), but much less than that reported in some studies conducted in other countries, e.g., 84.0% in Mexico City (Flores-Urbán et al., 2014), 81.3% in Korea (Park et al., 2018), and 80.0% in Glasgow (Altayar and Sutherland, 2006). These results indicate that *B. cereus* is common in lettuce. Emetic syndrome and diarrheal diseases are often associated with *B. cereus* counts of at least 10^5^ CFU/g (Osimani et al., 2018). In our study, concentrations below 10^5^ CUF/g were found in all lettuce samples. Thus, the loads at all levels were considered to be safe for consumption.

Multilocus sequence typing was used to evaluate the evolution and population diversity of *B. cereus*. The 100 *B. cereus* isolates from 46 lettuce farms represented 65 STs and seven CCs and were subtyped into five phylogenetic groups. ST233, ST378, ST1724, CC111, and CC142, which were crossed with different provinces, and each province had unique STs. Two strains isolated from lettuce and soil were assigned to ST26 and ST111, which were the same molecular types as those of the clinical isolates E6345 and F4794 (Hoffmaster et al., 2008). Eleven strains, except for those from pesticides, contained ST233, ST243, ST1001, ST1002, ST1210, and ST2171, which are listed as blood isolates in the *B. cereus* PubMLST database^3^. These results reveal a potential risk when consuming these lettuces directly or with minimal processing. ST1000 was the most frequent type in the 100 isolates and in lettuce, and ST177 was also a frequent type in lettuce, thus, these findings were different from the STs in vegetables on the Chinese market, in which ST26, ST770, and ST1605 were the most frequent types (Yu et al., 2019). Group IV was one of the three largest groups in this study, similar to its predominance in soil (Drewnowska et al., 2020). These results indicate the high genetic diversity of *B. cereus* isolates.

Soil and pesticides were the most frequently detected sources of *B. cereus* in our study, but the concentration in soil was higher than that in pesticides. In total, 7.3% of the soil samples had concentrations ranging from 2 × 10^4^ to 5 × 10^5^ CFU/g, which was consistent with previous studies (Drewnowska et al., 2020). Tracing analysis showed that soil, manure, pesticide and water had the same STs as lettuce, but soil and manure had more of these STs, indicating that *B. cereus* in lettuce was mainly from soil and manure. Additionally, the possible mechanism by which *B. cereus* spreads during lettuce planting were inferred as follows: soil-lettuce, manure-lettuce, pesticide-lettuce, manure-soil-lettuce, and water-manure-soil-lettuce. Quantitative microbial risk assessment of *E. coli* O157:H7 in lettuce revealed that bacterial concentration in soil, soil transfer by irrigation, and bacterial concentration in water were the most important input factors during lettuce preharvest (Pang et al., 2017; Bozkurt et al., 2021), which were partly consistent with our study and these indicated that the planting process needs to be controlled.

It has been demonstrated that *B. cereus* is the causative agent of two types of gastrointestinal diseases, namely, emetic syndrome and diarrheal syndrome (Osimani et al., 2018). The emetic form occurs due to a heat-stable toxin (cerulide) that is preformed in the food, while diarrhea is caused by the ingestion of viable cells, which produce enterotoxins in the small intestine (Osimani et al., 2018). In this study, in all the strains isolated from lettuce farms, only enterotoxins were detected, which was consistent with previous research (Park et al., 2018; Drewnowska et al., 2020).

Among the key toxin genes in *B. cereus* isolates, (1) the genes encoding the Nhe toxin were detected in 100.0% of the isolates in this study, especially *nheB*, which was distributed broadly. In addition, it has been shown that nearly 100.0% of *B. cereus* food poisoning outbreak strains harbor the Nhe toxin genes in Austria (Jessberger et al., 2019). (2) The genes encoding the Hbl toxin were present in 89.7% of the 68 isolates, which is somewhat higher than the value in Korea (Park et al., 2018), in which the *hblACD* genes were detected in 35.7% of lettuce isolates. In previous studies (Chon et al., 2015; Yu et al., 2019), the Hbl toxin genes were detected in 60.0% of *B. cereus* vegetable and soil isolates. (3) *EntFM* was detected in 91.2% of the strains, consistent with the results showing that 90.0 to 100.0% of *B. cereus* isolates harbored the *entFM* gene in *B. cereus* outbreak isolation (Jessberger et al., 2019). In total, 42.6% of the *B. cereus* isolates harbored the *cytK* gene. In Korea, 71.4% of the strains isolated from lettuce harbored *cytK* (Park et al., 2018), and *cytK* was present in 8.0 to 91.0% of the soil isolates (Drewnowska et al., 2020).

The enterotoxins Nhe and Hbl were detected by the Duopath^®^ Cereus Enterotoxins test. Nhe was detected in 100.0% of the 68 isolates, and Hbl was observed in 76.5% of the isolates, thus the rates were higher than those of dairy-associated isolates among which tested positive for Nhe and 30.8% for Hbl (Miller et al., 2018). Moreover, 92.0% of the lettuce isolates harbored Hbl. Additionally, in this study, we found that (1) Nhe was expressed when the *nheB* gene was present. (2) Hbl was detected when the strain harbored the *hblC* gene. The Duopath^®^ Cereus Enterotoxins test tracks Nhe by detecting the NheB component (encoded by *nheB*) in a sandwich complex with immobilized specific antibodies (Krause et al., 2010). NheA and NheC expression did not correlate with toxicity, while the expression of NheB showed a rough correlation with strain toxicity (Jessberger et al., 2019). In addition, the Duopath^®^ Cereus Enterotoxins test detected Hbl based on gold-labeled monoclonal antibodies of Hbl lytic component L2 (Hbl-L2; encoded by *hblC*).

Taken together, the results of the present study showed that despite the high presence of *B. cereus* in lettuce and farm environment samples, the loads were at levels considered to be safe for consumption. However, pathogenic STs (ST26 and ST111) were detected in lettuce and soil, and all *B. cereus* strains tested in this study carried at least 3 of the 8 enterotoxin genes. Nhe and Hbl enterotoxins were the major toxins among the *B. cereus* strains tested. Additionally, *B. cereus* present in planting environments (soil, manure, pesticide, and water) could be easily transferred to lettuce. Our study provides useful information for improving the microbiological safety of fresh lettuce.

## Data Availability Statement

The original contributions presented in the study are included in the article/supplementary material, further inquiries can be directed to the corresponding author/s.

## Author Contributions

YQ, YS, and CZ conceived and designed the experiments. YQ, CW, XD, XZ, QL, WW, YW, MG, and WT performed the experiments. YQ analyzed the data and wrote the draft. YB contributed in the reagents/materials/analysis tools. YS and CZ supervised the research. YS revised the manuscript. All authors contributed to the article and approved the submitted version.

## Conflict of Interest

YQ, CZ, and YS are employed by Institute for Agro-Food Standards and Testing Technology, Shanghai Academy of Agricultural Sciences, Shanghai, China and Shanghai Co-Elite Agro-Food Testing Service Co., Ltd. The remaining authors declare that the research was conducted in the absence of any commercial or financial relationships that could be construed as a potential conflict of interest.

## Publisher’s Note

All claims expressed in this article are solely those of the authors and do not necessarily represent those of their affiliated organizations, or those of the publisher, the editors and the reviewers. Any product that may be evaluated in this article, or claim that may be made by its manufacturer, is not guaranteed or endorsed by the publisher.

## Footnotes

1
https://pubmlst.org/bcereus/

2
https://www.ncbi.nlm.nih.gov/

3
https://pubmlst.org/bcereus/
